# Corticospinal Excitability during a Perspective Taking Task as Measured by TMS-Induced Motor Evoked Potentials

**DOI:** 10.3390/brainsci11040513

**Published:** 2021-04-18

**Authors:** Elizabeth Murray, Janet Brenya, Katherine Chavarria, Karen J. Kelly, Anjel Fierst, Nathira Ahmad, Caroline Anton, Layla Shaffer, Kairavi Kapila, Logan Driever, Kayla Weaver, Caroline Dial, Maya Crawford, Iso Hartman, Tommy Infantino, Fiona Butler, Abigail Straus, Shakeera L. Walker, Brianna Balugas, Mathew Pardillo, Briana Goncalves, Julian Paul Keenan

**Affiliations:** 1 JFK Neuroscience Institute, Hackensack Meridian Health and JFK Medical Center, Edison, NJ 08820, USA; elizabethm.murray@hackensackmeridian.org; 2 Cognitive Neuroimaging Laboratory, Montclair State University, Montclair, NJ 07043, USA; brenyaj1@mail.montclair.edu (J.B.); chavarriak2@mail.montclair.edu (K.C.); afierst23@mpsdnj.us (A.F.); ahmadn3@mail.montclair.edu (N.A.); canton23@mpsdnj.us (C.A.); hockeygirl330@gmail.com (L.S.); kkapila23@mpsdnj.us (K.K.); ldriever23@mpsdnj.us (L.D.); kayweaver16@gmail.com (K.W.); cdial21@mpsdnj.us (C.D.); ihartman23@mpsdnj.us (I.H.); tinfantino23@mpsdnj.us (T.I.); fbutler23@mpsdnj.us (F.B.); abigailcstraus@gmail.com (A.S.); balugasb1@mail.montclair.edu (B.B.); pardillom1@montclair.edu (M.P.); 3 Department of Psychology and Counseling, Georgian Court University, Lakewood, NJ 08701, USA; kkelly@georgian.edu; 4 Department of Biology, Northeastern University, Boston, MA 02115, USA; mayatcrawford@gmail.com; 5 Neuroscience and Cognitive Neuroscience Program, University of Maryland, College Park, MD 20742, USA; walkers8@terpmail.umd.edu; 6 School of Health and Medical Sciences, Seton Hall University, South Orange, NJ 07079, USA; briegoncalves@gmail.com

**Keywords:** perspective taking, self-awareness, self-representation, metarepresentation, theory of mind, transcranial magnetic stimulation

## Abstract

Only by understanding the ability to take a third-person perspective can we begin to elucidate the neural processes responsible for one’s inimitable conscious experience. The current study examined differences in hemispheric laterality during a first-person perspective (1PP) and third-person perspective (3PP) taking task, using transcranial magnetic stimulation (TMS). Participants were asked to take either the 1PP or 3PP when identifying the number of spheres in a virtual scene. During this task, single-pulse TMS was delivered to the motor cortex of both the left and right hemispheres of 10 healthy volunteers. Measures of TMS-induced motor-evoked potentials (MEPs) of the contralateral abductor pollicis brevis (APB) were employed as an indicator of lateralized cortical activation. The data suggest that the right hemisphere is more important in discriminating between 1PP and 3PP. These data add a novel method for determining perspective taking and add to the literature supporting the role of the right hemisphere in meta representation.

Perspective taking is a fundamental aspect of human existence and a likely driver of human brain evolution [1,2], including the enhancement of aspects of the frontal [3], and parietal lobes [4]. With the emergence of a variety of imaging techniques, a number of regions have been identified in perspective taking [5,6,7]. Across both patients [8,9], and experimental studies, converging evidence appears to implicate the right temporal parietal junction (rTPJ) [4,8,9], and medial prefrontal cortex (MPFC) [10,11,12], in adopting another person’s perspective.

One of the essential features of consciousness is perspective [13,14]. At the most basic level, all mammals possess a first person-perspective (1PP), also termed “central-representation” or “primary representation” [15]. This is the non-reflexive ability to simply know without explicit reflection or meta-representation of any kind. During 1PP, one would not think, “I am here”, but rather just be here [16]. The second person-perspective (2PP) is commonly defined as the ability to monitor one’s own mental state in a self-representational capacity, otherwise known as being self-aware [17,18]. In doing so, one is able to attend to one’s own cognitions in a proprietary, self-reflective manner. Differentiating between 1PP and 2PP is dependent upon the ability to actively monitor or mentalize one’s thoughts in the past, present, and future (2PP) as opposed to mere present awareness (1PP). The third-person perspective (3PP) is taking another’s perspective into account and is commonly considered theory of mind (ToM) or the ability to theorize about others’ minds.

Previous research into the understanding of perspective taking has utilized linguistic paradigms (e.g., [19,20,21,22]), self-face [23,24,25,26,27,28], affective interpretation tasks (e.g., [29,30,31,32]), tactile tasks (e.g., [33,34]), and lesion studies (e.g., [35,36,37]), using both on-line and off-line approaches (e.g., [38]).

Evidence suggests that 2PP and 3PP may recruit similar cortical areas, lending support for an underlying neuroanatomical network that mirrors their applied functional similarity. A number of brain regions have been implicated in these meta-representational functions, including: the right prefrontal cortex (e.g., [20,35,39,40,41]), parietal regions (e.g., [13,42,43,44,45]), the medial prefrontal cortex (MPFC; e.g., [14,46,47,48,49,50,51,52,53,54]), orbitofrontal regions (e.g., [55,56,57,58]), and the posterior cingulate cortex (e.g., [44,59,60,61]).

It remains unclear whether 1PP relies on similar or disparate cortical regions compared to those involved during 3PP and if 1PP is preferentially lateralized in the RH. One way in which 1PP has been successfully studied is through visio-spatial tasks which require the centering of one’s experiential space upon one’s self, which creates an egocentric reference space [15]. Vogeley et al., [15] created a visio-spatial paradigm in which the individual is required to shift between one’s own body axis perspective (1PP) and taking another’s vantage point as their own (3PP).

## 1. Motor Evoked Potentials (MEPs)

Transcranial magnetic stimulation (TMS) delivered to the “hand area” of the motor cortex elicits a motor-evoked potential (MEP) in the contra-lateral digits [62,63]. MEPs have become a part of almost every TMS application as they are used to measure individual differences in motor threshold (MT: [64,65], and it is generally thought unsafe to use TMS without gauging some aspect of MT [66,67,68,69]. Since its inception, MEPs have been used for wide-ranging investigations including post-stroke recovery [70], ALS [71], schizophrenia [72], intrinsic brain rhythm activity [73], and even veterinary medicine [74]. 

In terms of perspective taking, a study examined piano players who were presented with music they had practiced previously. When they thought the left hand part of the music was being played by another person, the MEPs in the left arm were greater, and MEPs increased as the participant’s empathy increased [75]. Further, previous work by our lab demonstrated that adopting another’s perspective (e.g., pretending to be a fan of an opposing sports team) led to greater left motor cortex/right hand MEPs [76].

Centered on first-person perspective, a number of researchers have employed TMS-induced motor-evoked potentials (MEPs) to measure lateralized cortical excitability during the presentation of self-descriptive adjectives [19]. The adjectives identified as highly or not at all descriptive of the individual resulted in increased right hemisphere excitability, indicating that the degree of self (including rejecting descriptions of oneself) could be discriminated via MEPs. The amount of one’s self-perception can alter MEPs such that participants’ positivity or sense of personal power results in differing senses of personal space [77].

Differences in perspective taking that exist in motor areas are not surprising. Lateralized hand response differences (e.g., reaction time and identification) exist such that there is a tendency for left-handed responses to be quicker for self-related stimuli [39,40,78,79]. Furthermore, the handedness of the individual plays a significant role in how self (compared to other) is processed in the brain. While right-handed individuals tend to be more consistent and the right hemisphere is dominant for self-processing, left-handed individuals display greater variability (and more left hemisphere involvement) in cortical response [80]. Therefore, both the hand that performs the task and an individual’s hand dominance influence perspective taking.

In order to further our understanding of the cortical mechanisms involved during 1PP and 3PP, we employed the same task in which participants were presented with virtual scenes of an avatar (i.e., a virtual character) and a number of red spheres [15]. Participants were instructed to report how many red balls would be visible either from their own (1PP) or the avatar’s perspective (3PP). The current study administered TMS to both the right and left motor cortices (MC) to determine the degree of lateralization during 1PP and 3PP. It was predicted that TMS administration to the right MC would generate larger MEPs during assumption of the avatar’s perspective (3PP). This prediction is suggestive of the greater involvement of the RH during ToM, lending support to the theoretical, anatomical and cognitive similarity between 2PP and 3PP [23,81]. The advantage of MEPs over traditional neuroimaging is the direct assay of excitability rather than the possibility that increased signal may be indicating inhibitory firings [65]. Therefore, if differences are found, a more direct interpretation is possible.

## 2. Materials and Methods

### 2.1. Participants

Ten right-handed Caucasian adults (4 men, 6 women; handedness was assessed using the Edinburgh Handedness Inventory) were recruited via flyer and word of mouth from Montclair State University and Seton Hall University (for similar samples, see [63,82,83]). The mean age of the participants was 22.1 (SD = 2.84) and all had a least some college education. Participants were appropriately screened using the TMS safety guidelines established by Wasserman [68,84]. All subjects received $25 for participation in the study and were treated in accordance with the standards and guidelines set forth by the Institutional Review Board (IRB) of Montclair State University. Written informed consent was obtained from all subjects. (IRB code: MSU IRB 424)

### 2.2. Materials

A TMS-Magstim 200 MonoPulse device with a 70 mm figure-8 coil was used to stimulate cortical areas of the brain. Stimuli were presented using SuperLab (Cedrus Corporation, Los Angeles, CA) on a Dell computer with a 17” inch CRT monitor. MEPs were acquired using Biopac MP150 amplifiers and accompanying acquisition software installed on a Dell computer. MEPs were recorded using three surface electrodes attached to areas of the hand, using EC2 electrode paste and surgical tape.

### 2.3. Procedure

For each subject, three surface electrodes were affixed to both hands, at the abductor pollicis brevis (APB) and the belly-tendon montage. A ground electrode was placed on the back of the wrist. Subjects were fitted with earplugs and a swim cap and then seated in front of a computer monitor with their head in a chin rest, 30 inches away from the computer monitor. Due to individual differences in corticoexcitability, motor threshold (MT) was first established. The MT was determined by stimulating the area of the primary motor cortex (M1) responsible for hand movement. The motor threshold is achieved by slowly increasing the stimulation intensity until hand movements (a) can be visually detected, in the contralateral hand, in 5/10 cortical stimulations (Wasserman [68,85]), and (b) met the IFCN guideline of MEPs over 50 [86]. MT determination was established for both hemispheres, for each subject.

**Figure 1:**
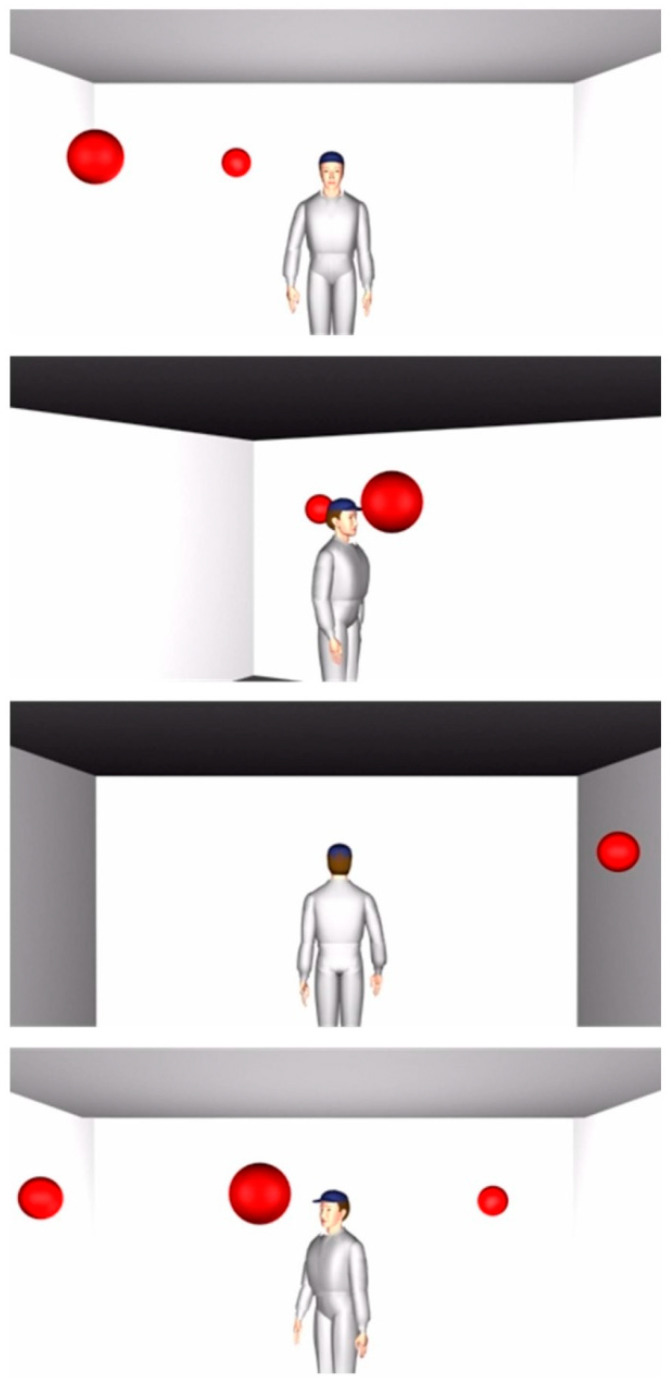
Stimuli of avatar and spherical balls. Each frame presented here demonstrates a different correct response for 1PP and 3PP. Stimuli were adapted from [15,87].

Subjects were then presented with a virtual scene that included an avatar and a varying number (1–3) of red spheres within or out of sight of the avatar (Figure 1). The subjects were asked to determine “how many balls they see” (1PP) or “how many balls the avatar sees” (3PP; [15]). These instructions were given verbally before each block. The experimenter recorded verbal responses. Single-pulse TMS was administered to the motor cortex of either the left or right hemisphere 150 ms or 300 ms following stimulus presentation onset. All stimulation was delivered at 100% MT due to IRB regulations at MSU which capped TMS at 100%. In each condition and for each hemisphere, 48 trials were presented (left hemisphere, 1PP; left hemisphere, 3PP; right hemisphere, 1PP; and right hemisphere, 3PP; 192 total pulses per individual were given). All stimuli remained on the screen until the participant made a verbal response. Reaction times were not recorded (that is, onset of verbal response time). Trials were separated by an inter-trial interval (ITI) of 1500 ms between each trial within condition. The left and right hemispheres were stimulated separately with the order of stimulation and conditions counterbalanced across subjects. TMS onset post-stimulus presentation was randomized for each condition.

Measures of TMS-induced MEPs of the APB were recorded. The EMG signal was amplified by a factor of 1000, filtered (bandpass amplifier filter between 1–500 Hz) and digitized using a sampling rate of 500 samples per second. All data were stored on a computer for off-line analysis. MEP data were filtered off-line using a Finite Impulse Response (FIR) linear bandpass filter (between 10–250 Hz) employing BIOPAC provided software. The remaining data were then rectified and averaged within-subject by condition. The threshold for data rejection was defined as baseline amplitudes that exceeded 100 μv. Following data rejection, group means were computed.

## 3. Results

**Figure 2:**
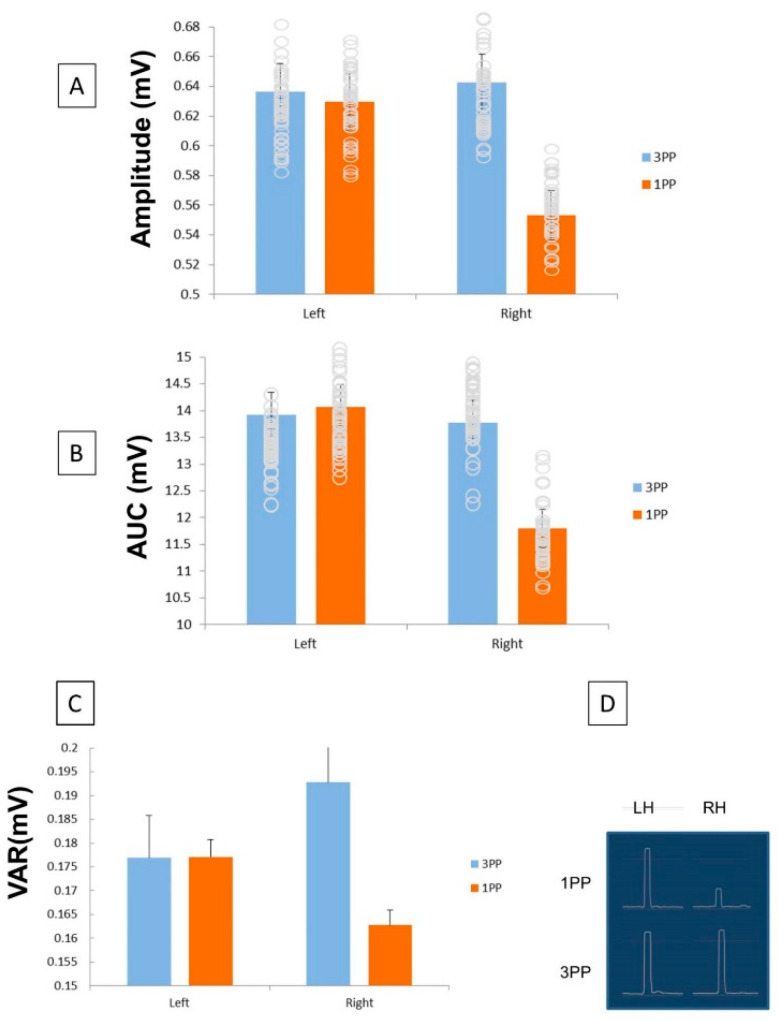
The peak (**A**), AUC (area under the curve) (**B**) and SD (standard deviation) (**C**) across Hemisphere for 1PP and 3PP. In all 3 measures, the 1PP Right Hemisphere condition differed significantly from all other conditions (all *p* < 0.05). All other comparisons were non-significant. A representative rectified, smoothed MEP is given for the 1PP (**D**) for RH and LH.

For each condition (1PP and 3PP), measures of TMS-induced MEPs for grand-averaged data were analyzed in terms of peak amplitude, area under the curve (AUC) and overall variability (SD). We began our analyses by directly testing a number of a priori comparisons. The timing of TMS onset was first analyzed using an independent samples t-test. Across conditions, the TMS pulse onset (150 vs 300 msec) did not impact MEP peak, AUC or variability (*p* > 0.05). As such, pulse onset was collapsed across all trials and conditions. A 2 × 2 × 2 (1PP/3PP; Left/Right Hemisphere Stimulation; 150/300 msec TMS-Onset) repeated measures analysis of variance (ANOVA) was performed. In the absence of a 3-way interaction (F (1,23) = 0.72, *p* > 0.05), a significant interaction between Hemisphere x Perspective was found (F (1,23) = 6.55, *p* < 0.02). The Right Hemisphere × 1PP differed significantly from all other conditions. 1PP, during right hemisphere stimulation, resulted in a significant decrease in peak amplitudes when compared to all other conditions. Additionally, a significant main effect for Perspective was found (F (1,23) = 5.57, *p* < 0.05), in that 1PP yielded less robust peak amplitudes as compared to 3PP (Figure 2). There were no other significant main effects (*p* > 0.05).

A second repeated-measures ANOVA was calculated to examine AUC differences. There was no significant 3-way interaction (F (1,23) = 0.02, *p* > 05). However, a significant interaction between Hemisphere x Perspective was found (F (1,23) = 11.63, *p* = 0.002). A significant main effect was found for the 1PP/3PP condition (F (1,23) = 8.029, *p* < 0.05), revealing a decrease in MEP AUC during the 1PP condition. A main effect for Hemisphere was also revealed (F (1,23) = 6.63, *p* < 0.05), such that the LH AUC was significantly greater than the RH AUC. The interaction between Hemisphere and Perspective, for both peak amplitude and AUC, indicates decreased right hemisphere activation during 1PP only.

Lateralized differences in MEP variability may offer unique insights into the consistent nature of the cortical response during differing perspectives. We therefore examined differences in SD using ANOVAs. There was no significant 3-way interaction (F (1,23) = 0.004, *p* > 05). There was no interaction between TMS Onset and Hemisphere or TMS Onset and Perspective (*p* > 0.05); however, a significant interaction between Hemisphere and Perspective was found (F (1,23) = 8.86, *p* < 0.007). Using the Bonferroni correction for multiple comparisons, post-hoc analysis revealed that the variability of the 1PP-right hemisphere condition was significantly lower compared to all other conditions (*p* < 0.05). Additionally, a significant main effect for Perspective was found (F (1,23) = 11.66, *p* < 0.002), such that the 1PP-condition was less variable than the 3PP-condition. Main effects for Hemisphere and TMS onset were not found (*p* >0.05).

## 4. Discussion

The current study sought to identify lateralized differences during first- and third-person perspective-taking. These data revealed significant differences within the RH for perspective taking. Both peak amplitude and AUC differed significantly between perspectives within the RH. No such differences were observed in the LH. An MEP reduction (for both amplitude and AUC) was noted for the RH during primary-representation, indicating that 1PP may require less activation in the RH. While it is not surprising to see a general decrease in MEP measures from 3PP to 1PP, as a function of task difficulty, the RH is considered to be dominant in spatial processing. In keeping with this, the LH did not evidence a decrease in MEP measures across perspectives.

The question remains: if meta-representational abilities of 1PP and 3PP seem to be lateralized in the RH, why was no significant difference in corticoexcitability between hemispheres during the 3PP condition found? There are a few possible explanations. First, although not significant, the RH did produce larger peak amplitudes than the LH during 3PP. However, the AUC measures were more similar. This may suggest inherent differences in MEP latency and length. As such, some studies indicate that the analysis of the MEP post-silent period (PMSP; [88]), may provide an alternate means of interpretation. However, these analyses were not possible with the data we collected, because our MEP recordings were not long enough to capture the inhibitory response (i.e., typically 300 ms). Furthermore, there is some evidence to suggest that the left motor cortex produces a greater MEP response as a function of greater activation of the left motor cortex in general [88]. 

Previously [89], increases in MEPs were found for self-related processing. Their results, and we assume ours, likely represent differences in TPJ functioning during self (1&2PP) and other (3PP) tasks. Bukowski [90] reviewed the perspective-taking literature and determined the singular region of interest across numerous methodologies was TPJ as a mediator of self–other differences in perspective. Noted, however, were the large number of studies in non-agreement including those employing TMS and tDCS.

Therefore, it is possible that reduced MEPs may indicate a region is particularly adept or specialized at processing stimuli. As we have previously reported [76], during a linguistic processing task, we found reductions in cortical activation during ToM tasks as ToM ability increases. Therefore, the possibility is likely more than speculative [91]. 

The current study further supports lateralized findings by demonstrating that self/other discriminations are significantly different between the hemispheres. These data add to a growing amount of evidence that the RH appears critical in evaluating self/other differences, tested across a number of different modalities in non-patient [28,80,92,93,94,95,96,97,98,99,100,101,102,103,104], and patient populations [35,105,106,107,108,109]. Decety and Lamm [110], have suggested that superior right parietal processing may be critical for both switching and differentiating self/other distinctions. Our current data support this possibility.

An underused measure of MEPs has been variability between responses, and here we show that the right MC has less variability in response in the 1PP condition. While it remains unclear what this may mean, we suggest that intuitively reduced variability may indicate increased efficiency. Typically, MEP variability is looked at in terms of either population differences [111], or physiological changes [112]. We suspect that a further use may be in terms of efficiency, though we admit this is speculative. We believe that testing other paradigms, in particular those that simulate “real-life” situations [113], such as knowing what another driver sees vs. what one sees, would be a valuable line of investigation. Future studies should also examine the associate priming [114], and we believe that a well-designed study could tease out both the ecological significance and the degree of semantic or associative priming the two tasks have.

Findings of this study should be interpreted with caution, due to the low number of participants (*n* = 10).

## 5. Conclusions

The ability to take different perspectives requires complex mental abilities. Testing participants by way of MEPS, we report that the RH serves a greater role in the discernment of 1PP and 3PP. In the LH, there was little difference in MEPs between 1PP and 3PP. However, the difference was significant between the perspectives in the RH. These results suggest that the RH is involved in meta-representation including perspective taking. 

## Data Availability

The data presented in this study are available on request from the corresponding author. The data are not publicly available due to privacy concerns.
